# Brain Tumor Classification Using Convolutional Neural Network and Bitterling Fish Optimization Algorithm

**DOI:** 10.3390/diagnostics16162659

**Published:** 2026-08-20

**Authors:** Hussein Sheet Ahmed Ahmed, Murat Yücel, Javad Rahebi

**Affiliations:** 1 Graduate School of Natural and Applied Sciences, Department of Electrical and Electronics Engineering, Gazi University, Ankara 06500, Turkey; hussein.sh.ahmed@uotelafer.edu.iq; 2 Department of Computer Science, Faculty of Education, University of Telafer, Nineveh 41016, Iraq; 3 Department of Electrical and Electronics Engineering, Gazi University, Ankara 06500, Turkey; muyucel@gazi.edu.tr; 4 Department of Electrical and Electronics Engineering, Istanbul Topkapi University, Istanbul 34662, Turkey

**Keywords:** bitterling fish optimization algorithms, brain tumor, classification, convolutional neural network

## Abstract

**Background/Objectives:** Brain tumor diagnosis using magnetic resonance imaging (MRI) plays an important role in clinical decision-making and treatment planning. However, manual interpretation of MRI scans is time-consuming and may result in variations among radiologists. This study aims to develop an automated multiclass brain tumor classification framework by integrating deep learning-based feature extraction with the Bitterling Fish Optimization (BFO) algorithm for effective feature selection. **Methods:** MRI images were first subjected to preprocessing to prepare them for deep learning analysis. Several pretrained convolutional neural network (CNN) architectures, including VGG16, VGG19, InceptionV3, ResNet50, EfficientNet, MobileNet, and ShuffleNet, were employed to extract informative deep features from the MRI images. The extracted features were then optimized using the BFO algorithm, which selected the most relevant features while reducing feature redundancy. The selected features were subsequently used for multiclass brain tumor classification. Model performance was evaluated using sensitivity, specificity, precision, accuracy, F1-score, and area under the curve (AUC). **Results:** The experimental results demonstrated that BFO-based feature selection improved the classification performance of the evaluated CNN architectures compared with their corresponding models without feature selection. Among the investigated CNN–optimizer combinations, the BFO-ShuffleNet framework achieved the best overall performance, obtaining 98.98% sensitivity, 98.99% specificity, 98.99% precision, 99.00% accuracy, and a 98.99% F1-score. These results indicate that BFO effectively identified the most discriminative features and enhanced the classification capability of the ShuffleNet architecture. **Conclusions:** The proposed deep learning and BFO-based framework provide an accurate and efficient approach for automated multiclass brain tumor classification from MRI images. The findings demonstrate that combining pretrained CNN models with BFO-based feature selection can reduce feature redundancy and improve diagnostic performance. In particular, BFO-ShuffleNet demonstrated the highest classification performance and shows considerable potential as a computer-aided diagnostic tool to support radiologists in brain tumor assessment and clinical decision-making.

## 1. Introduction

Brain tumors are a significant health concern, and need accurate diagnosis for effective treatment. Magnetic Resonance Imaging (MRI) is used to detect brain tumors because it provides detailed information about them. However, manual examination of MRI images takes a long time and can lead to diagnostic errors because of differences in image interpretation.

Recent advances in artificial intelligence (AI) have enhanced the automatic analysis of medical images. Also, deep learning approaches achieve good results in brain tumor classification. Convolutional neural networks (CNNs) can learn representative image features and have been applied to MRI-based diagnosis. Although CNN models often produce a large number of relevant features, this approach increases computational cost and reduces classification performance. To solve this problem, many studies have combined CNNs with optimization algorithms for feature selection. Common approaches include Particle Swarm Optimization (PSO), gray wolf optimization (GWO), the salp swarm algorithm (SSA), and other metaheuristic algorithms [1,2]. These methods improve feature selection, but the use of newer optimization algorithms for brain tumor classification is limited. Quantum computing (QC), machine learning (ML), and quantum machine learning (QML) are being explored in medical decision-making, with ML already widely used in medical imaging, disease prediction, and clinical decision support. QC and QML offer potential benefits for analyzing complex healthcare data and drug discovery, but their clinical use remains limited by current hardware, data, and integration challenges [3].

## 2. Literature Review

In ref. [4], the authors present an explainable deep learning framework dedicated to brain tumor detection based on a two-stage training approach. In the first step, a CNN is trained on the original MRI data to achieve a baseline. In the second phase, some explainable artificial intelligence (XAI) methods are employed for the generation of explanatory masks that highlight tumorous areas—specifically, LIME, Grad-CAM and SHAP. These masks are subsequently employed to augment the training data and retrain the CNN. The fused model using XAI-based data augmentation provides not only enhanced interpretability, but also improved classification performance. BFO-ShuffleNet is validated through tumor location, sensitivity, specificity, ROC-AUC and the explainability quality criteria of fidelity, stability and consistency, as well as its generalization over different datasets [4].

In ref. [5], a DL approach to a brain tumor detection system was proposed, working from MRI sequences. In the study, a dataset of large-scale brain tumors containing three classes—glioma, meningioma and pituitary tumors—was used. The authors used the YOLOv7 object detection model as their base model, and fine-tuned it with transfer learning to our medical signal domain. Some preprocessing was done to improve image quality and make it suitable for input into the network. The model was trained to optimize tumor presence and location detection on MRI scans. The CNS tumor detection ability was rated as high, but the model needed refinement in order to realize the possibility of accurate small-sized tumor detection.

In ref. [6], a DL- and optimization-based method is proposed for detecting brain tumors from MRI images. The images are firstly preprocessed with a mixed filter which is composed of Gaussian, mean and median filters. Subsequently, the tumors are segmented by means of thresholding and histogram methods. To obtain the texture features, the authors extract texture qualities and characteristics from the images after segmentation using a GLCM. Lastly, a gray wolf optimization algorithm (WOA)-patch-based convolutional neural network (CNN) is added into the workflow in order to classify the brain images as tumor-containing or non-tumor-containing.

Ref. [7] introduces a novel hybrid brain tumor segmentation model based on a 3D U-Net architecture. Multimodal MRI data are preprocessed using a six-step preprocessing pipeline including volume cropping, modality fusion, resampling, normalization and label reconstruction. A 3D Contextual Transformer (CoT) module is incorporated in the encoder–decoder framework to learn long-range contextual information. Skip connections are fused with Double-Attention (DA) blocks to help better encode features spatially and channel-wisely.

A hybrid model similar to Vision Transformer (ViT) and a Gated Recurrent Unit (GRU) is proposed in [8] for brain tumor detection. The spatial features were generated from MRI images by using the ViT model, and the dependencies between these features were learned by a GRU network. The class imbalance was adjusted by processing the dataset, and the model was trained with SGD, Adam, and AdamW optimizers under 10-fold cross-validation. The authors also improved model interpretability through explainable AI (XAI) techniques such as Attention Maps, SHAP, and LIME.

In ref. [9], classification of brain tumors on MRI was conducted with Vision Transformer (ViT)-based deep feature extraction and classical machine learning classifiers. The MRI images were initially preprocessed with the ViT model to extract complex and long-range spatial dependencies. These deep features from the ViT network were then utilized to train classic classifiers, e.g., Support Vector Machines (SVMs), Decision Trees and k-Nearest Neighbors (k-NN), to make classifications. This hybrid scheme combines the expressiveness of deep learning and simplicity of classical classifiers, showing stronger discriminability than using ViT directly for tumor detection.

In ref. [10] a customized CNN is proposed for brain tumor detection from MRI images. The model was trained on the BR35H dataset, which contains tumorous and non-tumorous MRI scans. To enhance transparency and interpretability, SHAP, LIME, and Grad-CAM explainable AI techniques were integrated into the framework. This XAI-enhanced CNN approach improved both diagnostic accuracy and clinician trust in AI-based medical decision systems.

In ref. [11], the authors present a brain tumor classification framework based on transfer learning using a pretrained EfficientNet V2 (B0–B3) model. Brain MRI images were categorized into four classes (glioma, meningioma, pituitary, and normal), and the final layer of the network was fine-tuned to match the target class. The model was trained using the Adam optimizer and categorical cross-entropy losses, following dataset randomization and a preprocessing step. Model performances were evaluated through accuracy, loss curve, and confusion matrix analyses to assess classification efficacy across tumor types.

In ref. [12], the authors propose HART-UNet, a Hybrid Attention–Residual U-Net architecture enhanced with Transformer blocks for more accurate brain tumor segmentation. The model integrates spatial self-attention, deep residual connection, and ResNet50 pretrained weighting to improve feature extraction ability. HART-UNet was trained on the BRATS’20 dataset and was validated on the Kaggle LGG and BTCpostop datasets. The framework effectively addresses both pre-operative and post-operative MRI segmentation challenges, including small residual tumor detection, showing it can handle difficult cases in clinical scenarios.

In ref. [13], the authors propose a Domain-Adapted nnU-Net (DA-nnUNet) model for unsupervised domain adaptation from adult to pediatric glioma segmentation. A domain classifier with a Gradient Reversal Layer (GRL) is integrated into an nnU-Net backbone to learn domain-invariant features without using any annotations from the pediatric domain. The model was trained on BraTS-Adult data and was evaluated on BraTS-PED, addressing the domain shift caused by imaging and clinical differences. This approach enabled effective pediatric glioma segmentation while also preserving the source-domain segmentation performance.

In ref. [14], the authors propose a Dilated Parallel Deep Convolutional Neural Network (PDCNN) for MRI-based brain tumor classification. The architecture employs two parallel convolutional paths with different dilation rates to capture both global (coarse) and local (fine) features while also mitigating gridding artifacts. Multiple preprocessing techniques were applied to the MRI images, and an average ensemble strategy was used to enhance the model’s robustness. The model was evaluated on three MRI datasets and showed high classification accuracy across both binary and multiclass tumor classification tasks.

In ref. [15], the authors propose a hybrid MobileNetV2–Support Vector Machine (SVM) model for brain tumor classification from MRI images. MobileNetV2 is employed as a lightweight feature extractor to obtain discriminative deep features, while an SVM is used for final classification. The model was trained and validated on the Kaggle MRI brain tumor dataset containing four classes: gliomas, meningiomas, pituitary tumors, and no tumors. This hybrid design achieved high accuracy while maintaining low computational complexity, making it suitable for many clinical applications.

In ref. [16], the authors propose SwinResDual (SwRD), a parallel hybrid architecture that integrates ResNet50 and Swin Transformer for brain tumor classification from MRI images. The model processes input images simultaneously through the CNN and Transformer branch to capture local spatial features and global contextual information. Features extracted from both branches are fused and passed to a linear classifier for final predictions. The framework was trained on a large-scale augmented MRI dataset for both multiclass and binary classification tasks, and enabled more accurate tumor diagnosis.

In ref. [17], the authors propose a CNN-based brain tumor classification framework using the EfficientNetV2-B0 architecture with transfer learning. Pretrained weights were utilized to extract discriminative features from MRI images, enabling efficient learning in spite of the limited medical data. The model was trained using data preprocessing and augmentation techniques to enhance its robustness and generalization ability. This approach achieved high classification accuracy while reducing training time, making it suitable for automated clinical diagnosis.

In ref. [18], the authors propose a hybrid DenseTransformer model that combines transfer learning and attention mechanisms for brain tumor classification from MRI images. Deep features extracted from a pretrained DenseNet201 model are integrated with a Transformer-based architecture and enhanced by Multi-Head Self-Attention (MHSA) and Squeeze-and-Excitation Attention (SEA) blocks. The framework was trained on the Br35H MRI dataset and compared with multiple pretrained CNN models, including VGG19, InceptionV3, Xception, MobileNetV2, and ResNet50V2. Model reliability and interpretability were further strengthened using statistical validation tests and XAI techniques such as Grad-CAM and LIME.

In ref. [19], the authors introduce DSIT-UNet (Dual-Stream Iterative Transformer UNet) for accurate brain tumor segmentation. The framework combines Iterative Transformer (IT) modules with a dual-stream encoder–decoder architecture to capture both local detail and long-range dependences. A Transformed Spatial–Hybrid Attention Optimization (TSHAO) module is integrated to enhance multiscale feature interaction and global–local balance. The model was evaluated on the TCIA, BraTS2020, and BraTS2021 datasets, demonstrating robust and precise tumor boundary segmentation.

In ref. [20], the authors suggest a meta-transfer learning scheme on top of nnUNet for brain tumor segmentation in meningioma and metastasis scenarios. The model is pretrained in a meta-learning manner on data from various brain tumor cases. The study also proposes a meta-transfer learning framework for improving brain tumor segmentation beyond gliomas. The approach fine-tuned a pretrained nnUNet model, which was original trained on glioma data, by using datasets that contained meningiomas and metastases. By transferring the learned representations, the model increases its ability to generalize to under-represented tumor subtypes. This strategy leads to improved segmentation accuracy and more robustness across diverse brain tumor categories.

In ref. [1], the authors propose a hybrid brain tumor classification framework that integrates CNN deep feature extraction, LMNN metric learning, and swarm-intelligence optimization. Features extracted from multiple pretrained CNNs are refined using LMNN and optimized via PSO and GWO for compact and discriminative representation. The optimized features are then classified using KNN, SVM, ANN, and Random Forest, with the DenseNet201-LMNN-GWO-KNN (DenseWolf-K) configuration yielding the best performance. Model robustness and interpretability are enhanced through external dataset validation and XAI techniques, including feature ranking and occlusion sensitivity maps.

In ref. [21], the authors propose an automated CNN architecture design framework for MRI-based brain tumor classification using Particle Swarm Optimization (PSO). PSO is employed to optimize architectural parameters such as the number and type of layers, filter size, filter count, and the number of fully connected neurons. This automated search replaces manual trial-and-error design, enabling performance-driven and efficient model construction. The resulting PSO-optimized CNN achieved high classification accuracies and demonstrated the effectiveness of optimization-based architecture searches in medical imaging.

In ref. [22], the authors propose PSO-UNet, a hybrid brain tumor segmentation framework that integrates Particle Swarm Optimization (PSO) with the U-Net architecture. PSO is employed to automatically tune critical hyperparameters, including filter number, kernel size, and learning rate. The optimized U-Net model achieves high segmentation accuracy while maintaining a lightweight architecture with low computational cost. This automated optimization strategy enhances robustness and generalization across multimodal MRI data.

In ref. [23], the authors investigate CNN-based brain tumor detection models enhanced with metaheuristic optimization techniques. Three models were developed: a baseline CNN, a CNN optimized with the Genetic Algorithm (GA), and a CNN combined with the Slime Mold Algorithm (SMA) for hyperparameter tuning. Experiments were conducted on a TBP project dataset and seven public datasets to evaluate model robustness. The hybrid CNN + SMA framework demonstrated the most superior and consistent performance in binary tumor classification compared to the baseline and GA-optimized models.

In ref. [24], the authors propose a CNN-based brain tumor classification framework which is enhanced with an attention mechanism to emphasize more informative regions in MRI images. PSO is employed to automatically optimize the CNN hyperparameters, improving the model’s performance. The approach was evaluated on a four-class Kaggle MRI dataset, with different attention strategies compared with each other. Model robustness was further assessed by introducing salt-and-pepper noise at different levels to test the adaptability and stability of the BFO-ShuffleNet model.

In study [25], the authors propose a bio-inspired deep learning framework for brain tumor classification that integrates a CNN with optimization techniques. An enhanced salp swarm algorithm (SSA) is used to optimize CNN architecture parameters and perform feature selection, while a Kernel Extreme Learning Machine (KELM) is employed for the classification task. The model was trained and evaluated on the CE-MRI Figshare dataset containing meningioma, glioma and pituitary tumor images. This hybrid approach improved both classification accuracy and computational efficiency compared to baseline CNN model.

In ref. [26], the authors propose a clustering-based brain tumor segmentation method using a multidimensional PSO algorithm. The approach automatically determines the optimal number of segments by combining intensity and spatial information from multimodal MRI data. Initial PSO-based segmentation is further refined using a Random Forest Classifier (RFC) to improve the accuracy. The method was evaluated on the BraTS challenge MRI dataset, demonstrating effective segmentation with limited labeled data.

In ref. [2], the authors propose a hybrid 3D deep learning framework for brain tumor detection and segmentation from volumetric MRI data. The architecture combines a 3D Fully Convolutional Neural Network (3D-FCNN) with interval type-2 fuzzy weighting and a Hybrid Transit Search Optimization–Particle Swarm Optimization (TSO-PSO) algorithm. The fuzzy weighting mechanism reduces the number of trainable parameters and also accelerates the training process, while the hybrid optimizer improves learning stability and accuracy. The model was evaluated on the BraTS 2019–2021 dataset, demonstrating efficient and more precise 3D tumor segmentation and classification.

Also, Ullah, Khan, Khan, Choi, and Anwar (2022) [27] introduced a TumorResNet model incorporating TL, which was trained on the BTD-MRI dataset, achieving the highest accuracy of 99.33% in two-class classification. Gómez-Guzmán et al. (2023) [28] combined a generic CNN with six TL architectures on mixed datasets, reaching 97.12% accuracy in four-class classification. Velpula et al. (2025) [29] proposed an ensemble voting classifier with DenseNet-201, ResNet-101, and SqueezeNet, attaining 99.69% accuracy in multiclass tasks and 100% in binary settings. Batool and Byun (2025) [30] introduced a lightweight multi-path CNN with optimal feature selection, achieving 96.03% accuracy while reducing overfitting. Study [31] presented FoTNet, with frequency-based feature extraction and focal loss. It obtained 92.5% accuracy and an AUC of 0.9754 for brain tumor diagnosis. Other studies focused on specialized tasks. Singh et al. (2025) [32] proposed a content-based retrieval method. Gupta et al. (2025) [33] developed a segmentation method. Kanchanamala et al. (2025) [34] proposed a quantum-inspired hybrid model with 94% accuracy.

Transfer learning has become a common approach for brain tumor classification from MRI images. Kumar et al. used a ResNet-50-based CNN to classify benign and malignant tumors and reported accuracies of 99.30% and 98.40%, respectively. Their method also improved precision, recall, F1-score, and image fusion quality. The work, however, was limited to binary classification and did not address multiclass brain tumor recognition [35].

Khushi et al. evaluated several pretrained CNN architectures and found that a customized EfficientNetB7 produced the highest classification accuracy. The reported performance reached 98.97% overall accuracy and 99.09% under five-fold cross-validation. The experiments were carried out on a public Kaggle dataset, while validation on independent clinical data and analyses of computational cost and model interpretability were not included [36].

Sandhiya and Raja combined deep features extracted from InceptionV3 and DenseNet201 with radiomic features and used a PSO-KELM classifier for four-class brain tumor classification. Testing accuracies of 97.92% and 98.21% were obtained on two benchmark datasets. Although the reported results were competitive, the experiments were performed only on publicly available datasets [37].

Amjad et al. presented a review of recent machine learning and deep learning methods for brain tumor classification, segmentation, and genetic alteration prediction using MRI and histopathology images. The review compared existing approaches and discussed their performance and limitations, and the challenges associated with datasets, multimodal imaging, and molecular analysis [38].

Many studies have used metaheuristic algorithms such as PSO, GA, GWO, WOA, HHO, and SSA for deep feature selection or CNN optimization. These methods can improve classification by removing unnecessary features [1,2]. However, comparing their performance is not straightforward because different studies often use different datasets, CNN models, classifiers, and evaluation protocols.

In this study, BFO is used to select the most useful features extracted from pretrained CNN models. During the search, BFO updates the feature subsets based on better solutions. If a search step does not improve the result, new positions are explored. As the search continues, the search area becomes smaller, enabling the algorithm to refine the selected features.

All CNN–optimizer models used the same dataset, classifier, data partitions, performance measures, population size, and stopping criterion. Since the CNN backbone was different for each model, our analysis of the results compares the complete CNN–optimizer combinations rather than the optimization algorithms alone. This enabled us to keep the experimental setup consistent and make the comparison more reliable. In addition to classification accuracy, the number of selected features was also examined. The results reported here are specific to the dataset and settings used in this study. The proposed framework combines pretrained CNN feature extraction with BFO-based feature selection. Among the evaluated CNN–optimizer combinations, BFO-ShuffleNet achieved the best performance and was selected as the final model. Pretrained CNN models extract deep features from MRI images. BFO then selects the final feature subset for classification. In this paper, BFO is used to select informative features from pretrained CNN feature vectors, and its performance is evaluated with ShuffleNet for brain MRI classification. Performance is compared with several CNN–optimizer combinations under identical training, classification, and evaluation settings so that differences reflect the feature-selection strategy rather than changes in the experimental setup. The findings apply only to the MRI dataset, pretrained CNN models, classifier, and optimization settings considered in this study. The goal of this study is to improve classification performance and reduce computational complexity.

The main contributions are:

- Deep features are extracted from pretrained CNN models and refined using Bitterling Fish Optimization (BFO).
- A binary feature-selection scheme is used to reduce the feature set while maintaining classification performance.
- BFO–ShuffleNet is compared with PSO-Xception, WOA-MobileNet, GA-Inception, and GWO-EfficientNet. All CNN–optimizer models use the same dataset, classifier, data partitions, population size, and stopping criterion.
- The models are tested with stratified five-fold cross-validation, and the results are reported using confusion matrices, ROC curves, confidence intervals, and statistical tests.
- The relationship between the number of selected features and classification performance is examined.

## 3. Methodology

The framework includes four stages: image preprocessing, deep feature extraction, BFO-based feature selection, and multiclass classification. MRI images are resized and normalized before feature extraction. A pretrained CNN extracts the deep feature vector, and BFO selects the final feature subset. The classifier then assigns each image to one of four classes: glioma, meningioma, pituitary tumor, or no tumor.

### 3.1. Dataset, Preprocessing, and Experimental Protocol

A publicly available brain MRI dataset from Kaggle was used in this study. The dataset includes four classes: glioma, meningioma, pituitary tumor, and no tumor. Table 1 presents the dataset details and the experimental settings. Since patient-level information was not available, all analyses were conducted at the image level.

All MRI images were resized to 224 × 224 pixels to match the input size of the pretrained CNN models. Pixel values were normalized to the range [0, 1] by dividing them by 255, and the labels were converted into one-hot vectors. No additional preprocessing or image enhancement was applied.

Performance was evaluated using stratified five-fold cross-validation. In each fold, 80% of the images were used for training and 20% for validation, while preserving the original class distribution.

The experiments were performed in two stages. The first stage used VGG16, VGG19, InceptionV3, and ResNet50 to compare classification results before and after BFO feature selection. The second stage compared CNN–optimizer models built from Xception, MobileNet, Inception, EfficientNet, and ShuffleNet with different metaheuristic algorithms. The Bitterling Fish Optimization (BFO) algorithm was then used to select the most useful features. The selected features were used to train the classifier, and the validation images were used only for testing. The validation data were never included during feature selection or model training, which prevented information leakage.

### 3.2. Bitterling Fish Optimization (BFO)

The BFO algorithm is a bio-inspired optimization approach based on reproductive behavior of bitterling fish. Other fish species release their eggs into open water, but bitterling fish deposit their eggs inside freshwater mussels, so the eggs are protected during early development. Male fish compete to secure appropriate mussels, and female fish select mates based on their fitness. These behaviors inspire the exploration and exploitation mechanism of the optimization process.

**Figure 1:**
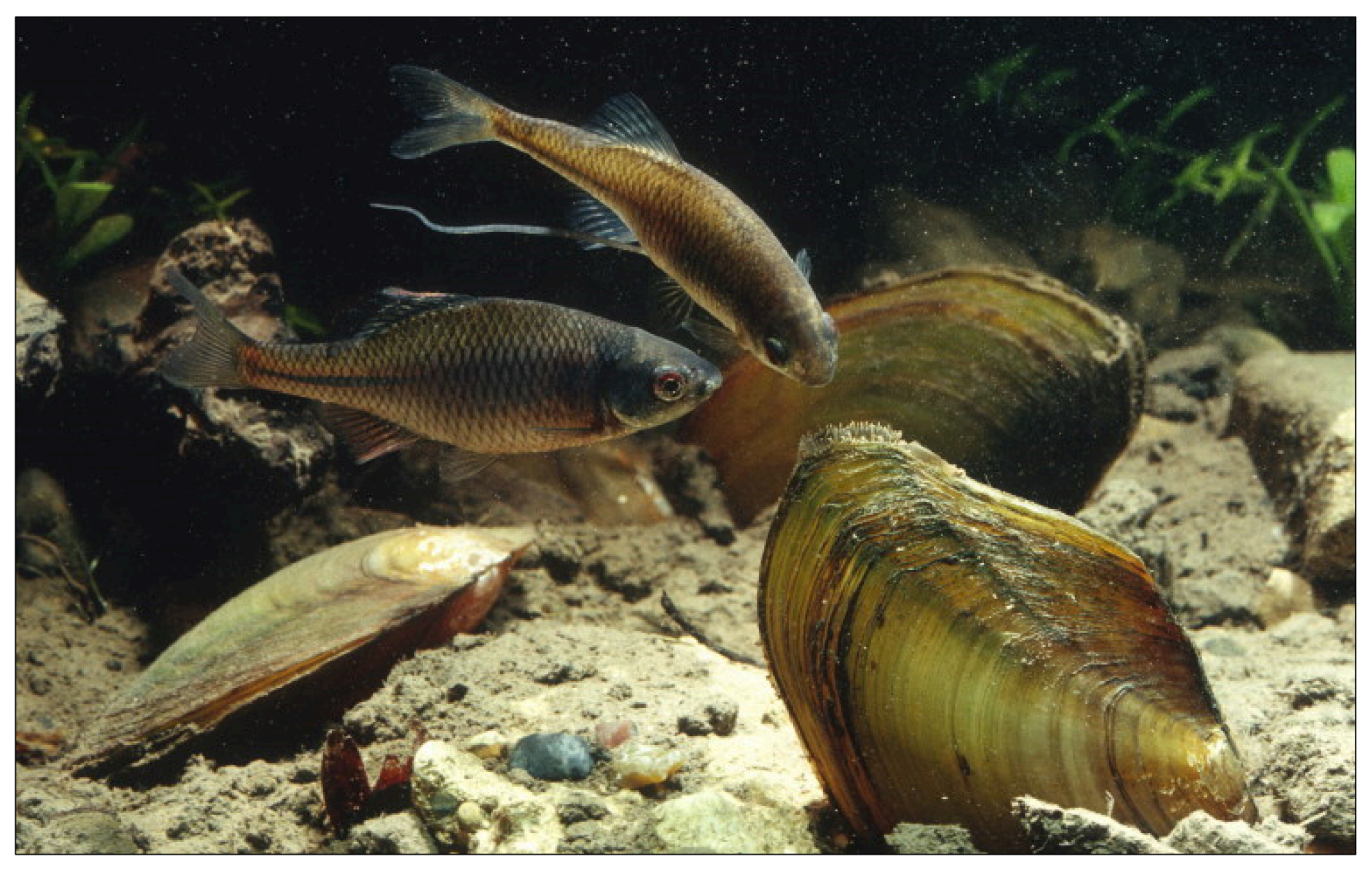
Biological inspiration of the BFO algorithm.

In the BFO algorithm, each fish represents a candidate solution, and its fitness shows the objective function. The interaction between fish and mussels models the search process. This means the candidate solutions are updated continuously to enhance their fitness. Therefore, the algorithm balances global exploration and local exploitation to find the optimal solution. Figure 1 shows the biological behavior that inspires the BFO algorithm.

The following assumptions are considered to model the BFO algorithm:

- Each candidate solution shows a bitterling fish.
- The quality of each candidate solution is evaluated by the objective function.
- Candidate solutions are produced randomly in the initial population.
- New candidate solutions are produced during local and global search.
- Poor candidate solutions are removed during the optimization process.

Each candidate solution is provided as a bitterling fish Equation (1). The initial population is produced randomly:

(1) $$F_{i}=[F_{i}^{1},F_{i}^{2},F_{i}^{3},\ldots ,F_{i}^{D}]$$

(2) $$F=\left[\begin{array}{cccc} F_{1}^{1} & F_{1}^{2} & \ldots & F_{1}^{D}\\ F_{2}^{1} & F_{2}^{2} & \ldots & F_{2}^{D}\\ \vdots & \vdots & \vdots & \vdots \\ F_{n}^{1} & F_{n}^{2} & \ldots & F_{n}^{D} \end{array}\right]$$

Here, D is the number of decision variables in each solution, and i is an index. The population is shown by matrix F, where $F_{i}^{j}$ is the $j_{t}$ decision variable of the $i_{t}$ solution. The initial population is created randomly within the lower and upper bounds [l, u], as shown in

(3) $$F_{i}^{j}=l+\left(u-l\right)\cdot r$$

where *r* is random between 1 and 0.

Each fish in the population represents one feature subset. The quality of each subset is measured by the objective function $f\left(F_{i}\right)$. This value is used to rank the population and update the search in the next iteration. For a population of $N_{p}$ fish, the fitness values are written as

(4) $$Fittness=\left[\begin{array}{cccc} {f(F}_{1}^{1} & F_{1}^{2} & \ldots & F_{1}^{D})\\ {f(F}_{2}^{1} & F_{2}^{2} & \ldots & F_{2}^{D})\\ \vdots & \vdots & \vdots & \vdots \\ f(F_{n}^{1} & F_{n}^{2} & \ldots & F_{n}^{D}) \end{array}\right]$$

where $f\left(F_{i}\right)$ is the objective-function value of the ith fish and $N_{p}$ is the population size. The optimization process favors solutions with better fitness values. In this study, the objective function measures the classification performance of the selected feature subset. Therefore, during each iteration, a solution updates its position by moving toward the current best solution or exploring another best solution in the population. Equation (5) shows this process:

(5) $$F_{i}^{t+1}=\left\{\begin{array}{c} \begin{array}{cc} {J\cdot F}_{i}^{t}+\left(F^{+}-J\cdot F_{i}^{t}\right)\cdot \delta & r\leq P \end{array}\\ J\cdot \begin{array}{cc} F_{i}^{t}+\left(F^{*}-{J\cdot F}_{i}^{t}\right)\cdot \delta & r>P \end{array} \end{array}\right.$$

In Equation (5), $F_{i}^{t}$ and $F_{i}^{t+1}$ represent the current and update positions of the $i_{t}$ solution, respectively. $F^{*}$ is the current best solution. $F^{+}$ is a randomly selected solution from the population. The random r and δ values are between zero and one. J controls the search step size and slowly decreases during the optimization process to shift the search from global exploration to local exploitation.

(6) $$J\left(t\right)=\left(J\left(1\right)-\frac{J\left(1\right)\cdot t}{Maxt}\right)\cdot U(t)$$

where $J\left(1\right)$ is the initial step size, t is the current iteration, and Maxt is the maximum iteration number. The random sequence U(t) is generated by Equation (7).

(7) $$U\left(t+1\right)=cos(t\times {cos}^{-1}(U(t)))$$

Combining Equations (6) and (7) results in Equation (8).

(8) $$J\left(t+1\right)=\left(J\left(1\right)-\frac{J\left(1\right)\cdot t}{Maxt}\right)\cdot cos(t\times {cos}^{-1}(U(t)))$$

The parameter P controls the probability of two search strategies in Equation (5). Its value reduces during the optimization process based on Equation (9).

(9) $$P=\left|1-\frac{t}{\sqrt{1+t^{2}}}\right|+\frac{rand}{t^{a}}$$

If the current search is unsuccessful, the candidate solution explores a new search area by the update rule:

(10) $$F_{i}^{t+1}=\left\{\begin{array}{c} \begin{array}{cc} {J\cdot F}_{i}^{t}+\left(F^{*}-J\cdot M\right)\cdot \delta & r\leq 0.5 \end{array}\\ \;l\begin{array}{cc} +\left(u-l\right)\cdot \delta & \;\;\;\;\;\;\;\;\;\;\;\;\;\;r>0.5 \end{array} \end{array}\right.$$

Here, M is the mean position of the population and it is calculated by

(11) $$M=\frac{\sum_{i=1}^{n}F_{i}^{t}}{n}$$

New candidate solutions are created around the current solution:

(12) $$F_{i}^{t+1}=F_{i}^{t}+R*rand(0,1)$$

where R is the search radius. Its value reduces during the optimization process to enhance local searching.

### 3.3. BFO-Based Feature Selection and Implementation Settings

#### 3.3.1. Feature Representation

The original BFO algorithm searches in a continuous space, whereas feature selection requires binary decisions. For this reason, each BFO solution is represented as a binary vector:

(13) $$z_{i}=\left[z_{i}^{1},z_{i}^{2},\ldots ,z_{i}^{D}\right]$$

where D is the number of deep features extracted from the selected CNN model. Each element is defined as

(14) $$z_{i}^{j}=\left\{\begin{array}{cc} 1, & \mathrm{if}\;\mathrm{feature}\;j\;\mathrm{is}\;\mathrm{selected}\\ 0, & \mathrm{otherwise} \end{array}\right.$$

A value of 1 keeps the feature, while 0 removes it. Let

(15) $$f=\left[f_{1},f_{2},\ldots ,f_{D}\right]$$

The selected feature subset is obtained by

(16) $$f_{i}^{*}=f⊙z_{i}$$

where $⊙z_{i}$ denotes element-wise multiplication. The resulting feature vector is

(17) $$f_{i}^{*}=\left[f_{1},z_{i}^{1},f_{2},z_{i}^{2}\ldots f_{D}z_{i}^{D}\right]$$

After each BFO position update, the continuous solution is converted into a binary vector using the selected binarization method. This step ensures that every solution represents a valid feature subset during optimization.

**Figure 2:**
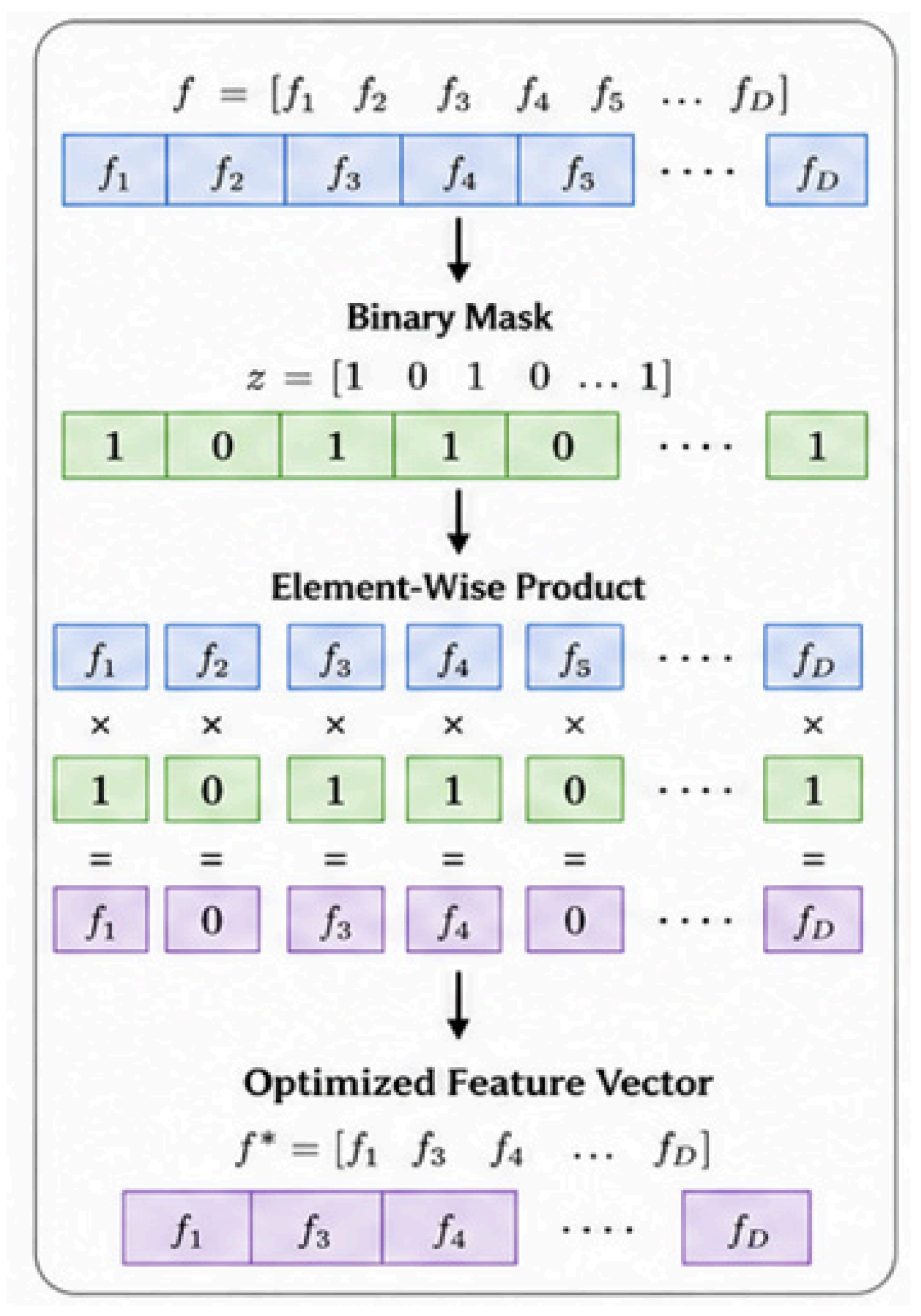
CNN feature selection using a binary feature mask in BFO.

Figure 2 shows how the original CNN feature vector is converted into the final feature subset selected by BFO.

#### 3.3.2. Fitness Function

Each BFO solution selects a subset of deep features from the pretrained CNN. These features are used to train the classifier, and the classification result is used to calculate the fitness value.

The fitness value ranks all solutions in the population. Better solutions are kept for the next iteration, while weaker ones are replaced. The fitness value is calculated for every solution at each iteration. All optimization algorithms use the same data folds, classifier, population size, fitness function, and stopping criterion.

The fitness function is based on two factors: classification error and the number of selected features. Let ($z_{i}$) be the binary feature mask, (D) the total number of extracted features, and ($|S_{i}|$) the number of features selected by ($z_{i}$). The objective function is

(18) $$J\left(z_{i}\right)=\alpha E\left(z_{i}\right)+\left(1-\alpha \right)\frac{|S_{i}|}{D}$$

where $E\left(z_{i}\right)$ is the classification error and (α in [0, 1]) controls the contribution of the two terms. The classification error is

(19) $$E\left(z_{i}\right)=1-{Acc}_{val}\left(z_{i}\right)$$

where ${Acc}_{val}\left(z_{i}\right)$ is the validation accuracy obtained from the selected features. Lower values of (J ($z_{i}$)) indicate better solutions because they combine higher classification accuracy with fewer selected features. The value of (α) was fixed at α = 0.95 for all folds and all optimization methods.

#### 3.3.3. Implementation Parameters

All optimization methods used the same training and validation folds, CNN feature extraction procedure, classifier, population size, maximum number of iterations, and stopping criterion. With these settings fixed, the comparison reflects the behavior of the optimization algorithms. The same random seed was used for all optimization algorithms.

Table 2 lists the implementation parameters of BFO and the other optimization algorithms. All optimization algorithms used the same parameter values. Each algorithm was run 30 times, and the reported results are the averages of these runs.

During the BFO search, the search step, search radius, and strategy probability were updated using Equations (6), (9) and (12). A threshold value of 0.5 converted the continuous search positions into binary feature masks. The same parameter values were used in all cross-validation folds and repeated runs.

#### 3.3.4. Stopping Criteria

The optimization ended after the maximum number of iterations was reached. At each iteration, the solutions were updated and ranked by their fitness values. The solution with the lowest objective value was selected as the final feature subset. All optimization algorithms used the same stopping criterion.

#### 3.3.5. Computational Complexity

The optimization requires $O\left(N_{p}\times T\times D\right)$ search operations. The total execution time also depends on the computational cost of training and validating the classifier. Most of the computation comes from evaluating the selected feature subsets during classifier training and validation. As a result, the total execution time depends on both the optimization algorithms and the computational cost of the classifier.

#### 3.3.6. Rationale for Selecting BFO

Algorithms such as PSO, GA, GWO, and WOA are commonly used for feature selection. BFO was selected because its search radius decreases during optimization. This enables wider exploration in the early iterations and a more focused search in the later iterations. This allows the algorithm to examine different feature combinations first and then focus on the most promising ones.

The search direction is updated from the fitness values of the current population, allowing the selected feature subset to improve over successive iterations.

BFO was evaluated together with PSO, GA, GWO, and WOA using the same preprocessing steps, CNN features, classifier, data folds, fitness function, and stopping criterion. All methods were evaluated under the same settings, so the comparison focuses on the optimization approach.

#### 3.3.7. Classification Model

For an input MRI image I , the pretrained CNN extracts a deep feature vector:

(20) $$f=\Phi_{k}\left(I\right)\in R^{D}$$

BFO applies a binary mask z* to select the final features:

(21) $$f^{*}=f⊙z^{*}$$

where ⊙ denotes element-wise multiplication.

The selected features are classified by a multiclass SVM. The predicted class label is

(22) $$ŷ={arg}_{mac}g_{c}f^{*}$$

where $g_{c}f^{*}$ is the decision function for class *c*.

**Figure 3:**
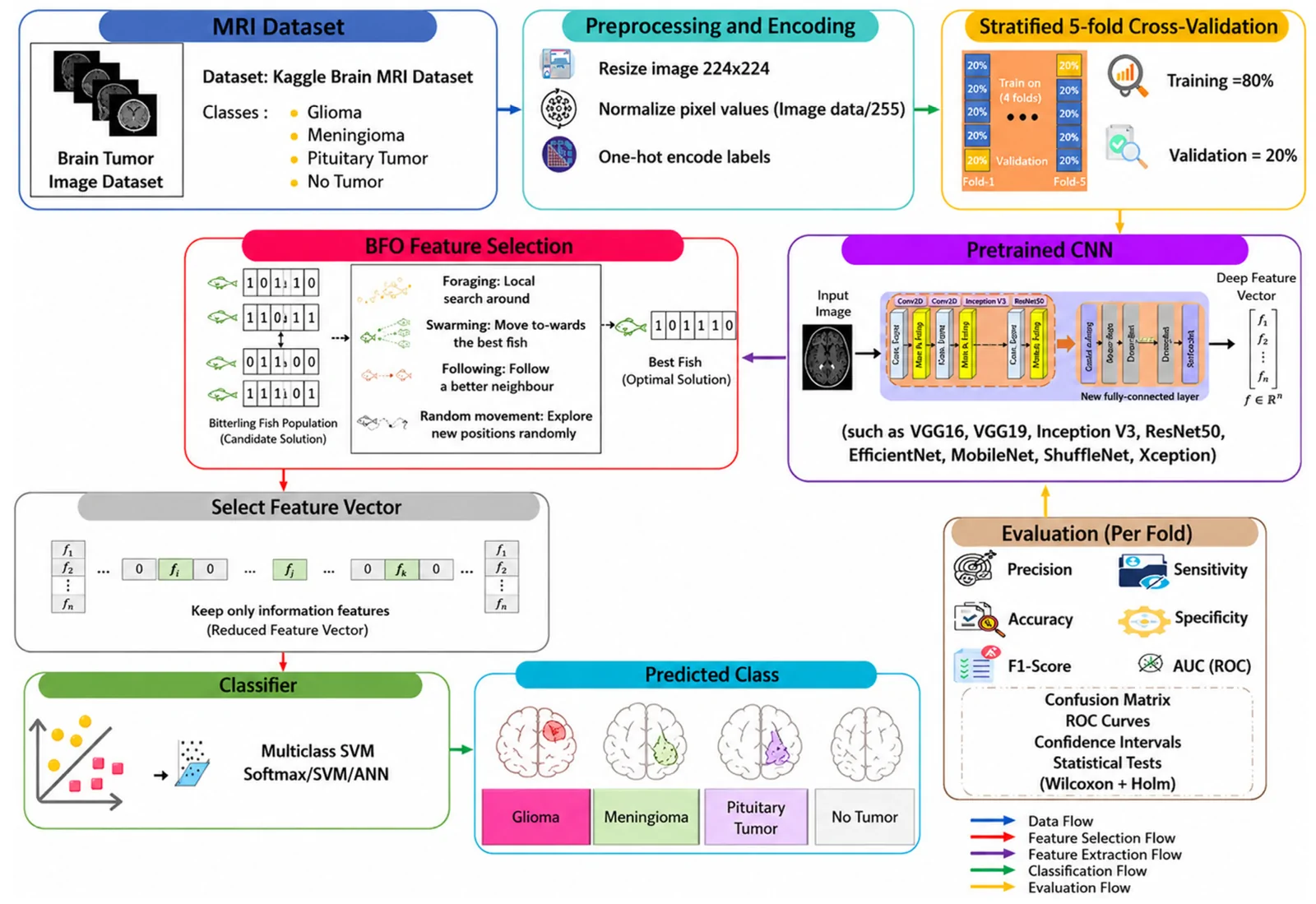
Workflow of the BFO-based brain tumor classification framework with stratified five-fold cross-validation.

Figure 3 shows the feature extraction process using pretrained CNN models. MRI images are preprocessed and normalized before being used as inputs for the networks. Then deep image features are extracted and passed to the subsequent feature-selection step.

## 4. Results and Discussion

A comprehensive analysis has been conducted to identify the optimal combination of approaches. Deep features were extracted from MRI images using pretrained CNN models. BFO was then used to select the final feature subset. The BFO approach was combined with a pretrained CNN to categorize the same datasets used in the first model. Accuracy and other crucial metrics are used to evaluate how well the approaches developed from the confusion matrix work. Metrics like total accuracy, sensitivity, specificity, precision, and F1-score were taken into account for multiclass classification. False Positive (FP), True Positive (TP), True Negative (TN), and False Negative (FN) are the basic terminology used in this analysis; they represent positive and negative classifications, respectively.

Accuracy (ACC), sensitivity, specificity, precision, and F1-score were all computed using these indications in the following ways:

(23) $$Accuracy=\frac{TP+TN}{TP+TN+FP+FN}\times 10$$

(24) $$Sensitivity=\frac{TP}{TP+FN}\times 100$$

(25) $$Specificity=\frac{TN}{TN+FP}\times 100\%$$

(26) $$Precision=\frac{TP}{TP+FP}\times 100\%$$

To compare various combinations and confirm the efficacy of the suggested technique, a number of scenarios were created and evaluated. The study included two sets of experiments. Deep features were extracted from VGG16, VGG19, InceptionV3, and ResNet50 to examine the performance of BFO with different pretrained CNN models. VGG16 features were also used to compare the classifiers and select the one used in the remaining experiments. The selected BFO-ShuffleNet model was then compared with PSO-Xception, WOA-MobileNet, GA-Inception, and GWO-EfficientNet under the same training and evaluation settings. VGG16 features were classified using ANN, SVM, Decision Tree, AdaBoost, Random Forest, and ensemble classifiers. Table 3 compares their performance.

**Figure 4:**
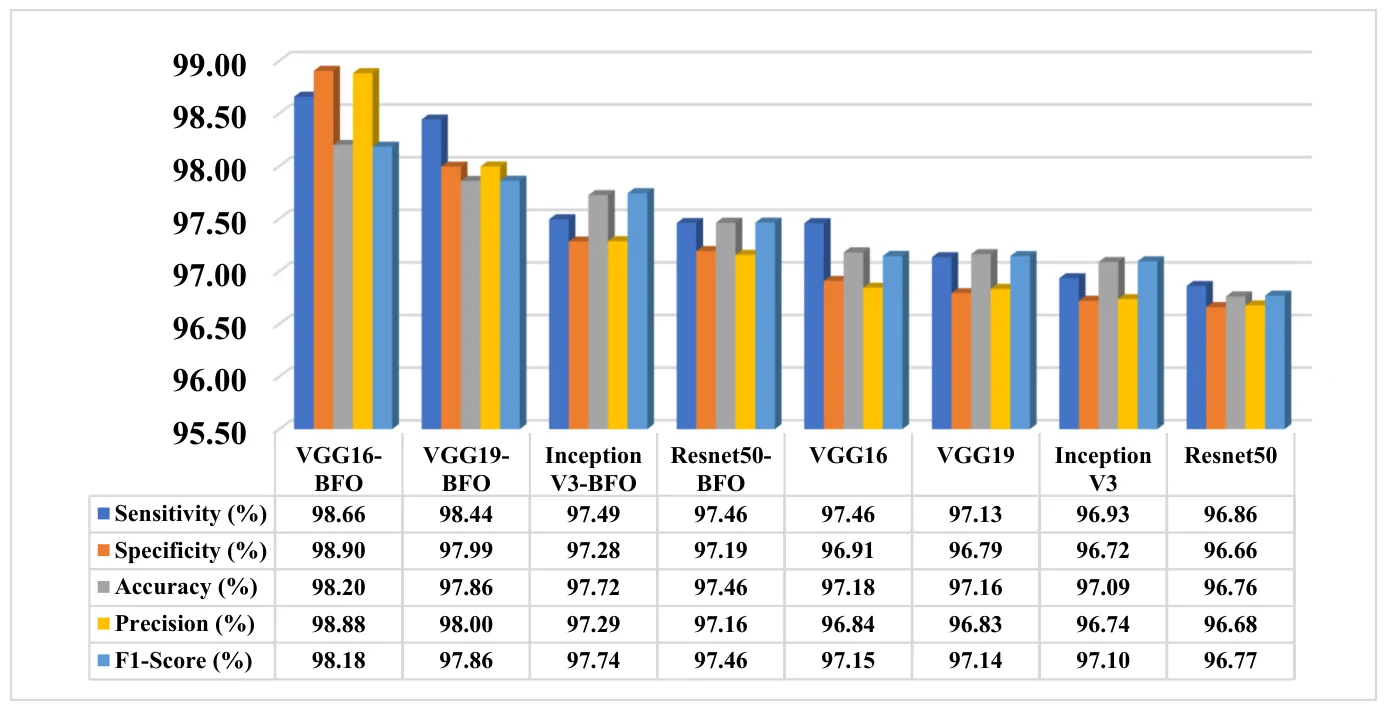
Performance comparison of VGG16, VGG19, InceptionV3, and ResNet50 before and after BFO feature selection.

Table 3 presents the performance of the evaluated classifiers using BFO-selected VGG16 features. SVM achieved the highest accuracy of 97.84%, with 98.37% specificity and 98.38% precision. It also completed the classification in 0.01 s. Based on this result, SVM was used in the subsequent CNN and feature-selection experiments. AdaBoost achieved an accuracy of 97.63%, followed by ANN at 97.55%, Ensemble at 97.31%, Decision Tree at 97.25%, and Random Forest at 97.17%. AdaBoost provided the highest sensitivity of 98.13%, while the Ensemble classifier required the longest processing time of 0.04 s. Figure 4 compares the performance of VGG16, VGG19, InceptionV3, and ResNet50, before and after using the BFO algorithm.

The evaluation was performed using sensitivity, specificity, accuracy, precision, and F1-score metrics. The results show that BFO enhances the performance of all CNN approaches. VGG16-BFO achieved the best results, with a sensitivity of 98.66%, specificity of 98.90%, accuracy of 98.20%, precision of 98.88%, and F1-score of 98.18%. The results show that BFO feature selection improved the classification performance of VGG16. The VGG19-BFO model achieved the second-best performance, with 97.86% accuracy, 98.44% sensitivity, and 98.00% precision. These results show that BFO improves the classification performance of both VGG-based models. InceptionV3-BFO reached 97.72% accuracy, 97.49% sensitivity, and 97.74% F1-score. ResNet50-BFO achieved 97.46% accuracy, 97.46% sensitivity, and 97.46% F1-score. Both models showed improved performance after applying BFO-based feature selection. BFO improved the classification accuracy of all four CNN models. VGG16 increased from 97.18% to 98.20%, VGG19 from 97.16% to 97.86%, InceptionV3 from 97.09% to 97.72%, and ResNet50 from 96.76% to 97.46%. The sensitivity of VGG16 also improved from 97.46% to 98.66%.

The non-optimized CNN models demonstrated relatively strong classification capabilities, with accuracies ranging from 96.76% to 97.18%. Among them, VGG16 achieved the best performance, followed closely by VGG19, InceptionV3, and ResNet50. However, all baseline models showed lower sensitivity, specificity, precision, and F1-scores compared to their BFO-enhanced counterparts, indicating the presence of redundant or less informative features that negatively affected classification performance.

**Figure 5:**
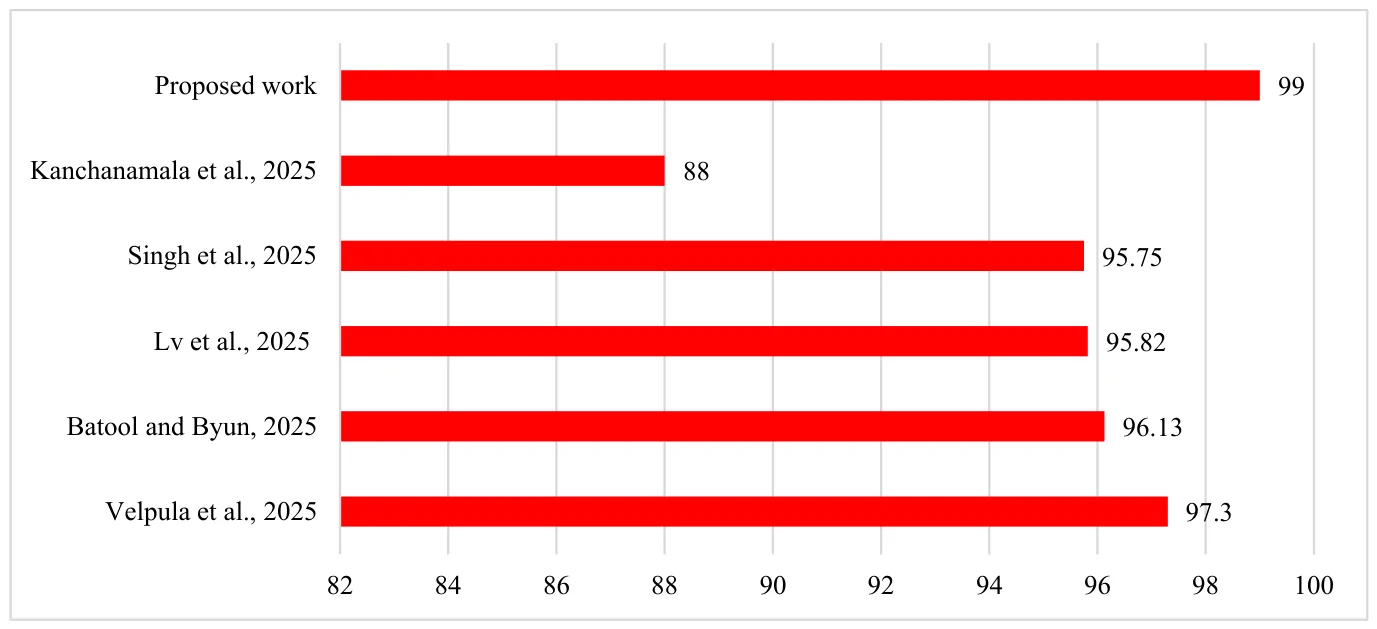
Accuracy comparison of the BFO-ShuffleNet model with previous studies by Velpula et al. [29], Batool and Byun [30], Lv et al. [31], Singh et al. [32], and Kanchanamala et al. [34].

Figure 5 compares the accuracy of the proposed BFO-ShuffleNet model with that reported in previous studies by Velpula et al. [29], Batool and Byun [30], Lv et al. [31], Singh et al. [32], and Kanchanamala et al. [34].

**Figure 6:**
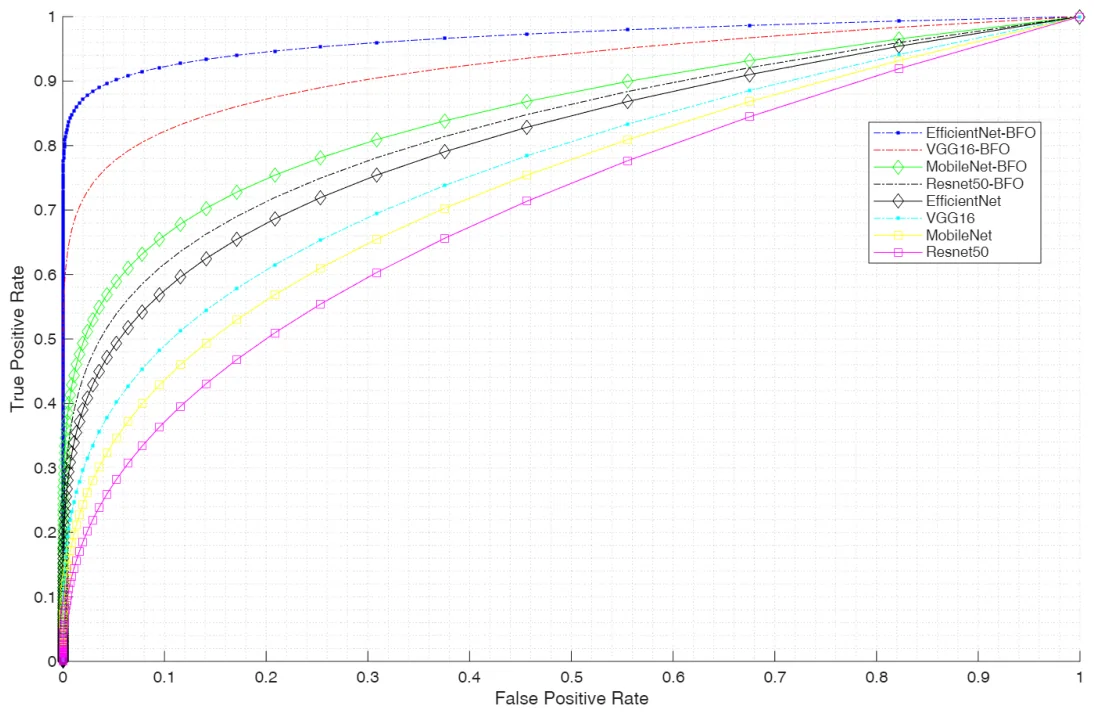
ROC diagram with AUC numerical results.

Figure 6 compares the one-versus-rest ROC curves of VGG16, VGG19, InceptionV3, and ResNet50 before and after BFO feature selection. For all four CNN models, feature selection increased both the ROC curves and the AUC values. Among the tested models, VGG16 combined with BFO gave the highest AUC. The results show that removing less informative deep features helped the CNN models distinguish the four brain MRI classes more effectively.

**Figure 7:**
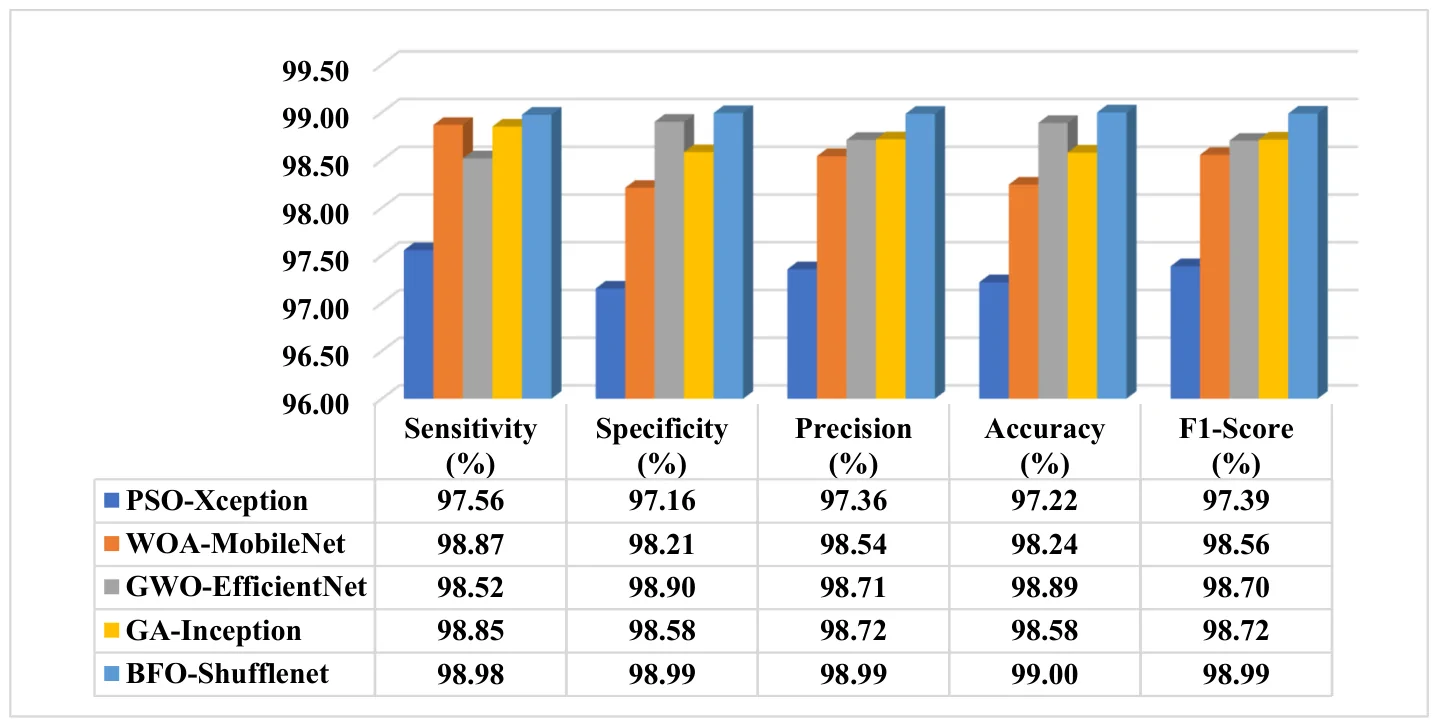
Performance comparison of PSO-Xception, WOA-MobileNet, GWO-EfficientNet, GA-Inception, and BFO-ShuffleNet.

Figure 7 compares the performance of five CNN–optimizer models: PSO-Xception, WOA-MobileNet, GWO-EfficientNet, GA-Inception, and BFO-ShuffleNet.

The models were evaluated using sensitivity, specificity, precision, accuracy, and F1-score metrics. All optimization approaches achieved high classification performance, with accuracy values above 97%. Among the evaluated approaches, BFO-ShuffleNet achieved the best overall performance, with a sensitivity of 98.98%, specificity of 98.99%, precision of 98.99%, accuracy of 99.00%, and F1-score of 98.99%. GWO-EfficientNet reached an accuracy of 98.89%. GA-Inception, WOA-MobileNet, and PSO-Xception achieved accuracies of 98.58%, 98.24%, and 97.22%, respectively. After BFO-ShuffleNet, WOA-MobileNet recorded the highest sensitivity (98.87%), while GWO-EfficientNet recorded the highest specificity (98.90%) among the remaining CNN–optimizer models. Table 4 presents the performance of the CNN–optimizer models. Each model uses a different pretrained CNN together with a metaheuristic feature-selection algorithm. All models were tested with the same dataset, classifier, data partitions, population size, and stopping criterion. Since the CNN model and the optimization algorithms differ, the results compare the complete CNN–optimizer models rather than the optimization algorithms alone.

Results are reported as mean ± standard deviation and 95% confidence intervals from repeated stratified five-fold cross-validation. Statistical differences between BFO-ShuffleNet and the other CNN–optimizer models were assessed with the two-sided Wilcoxon signed-rank test. Holm correction was used for multiple comparisons.

**Figure 8:**
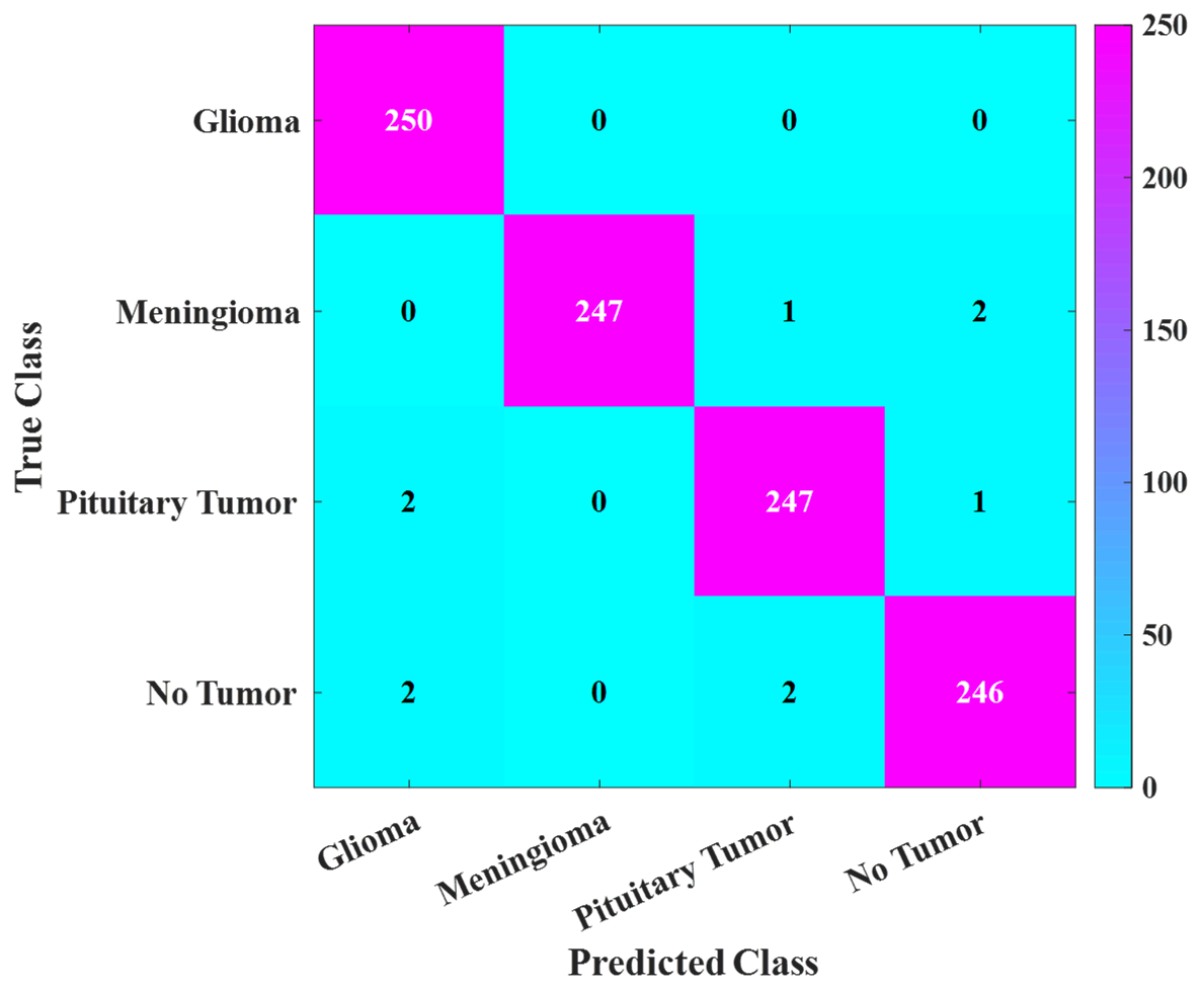
Overall confusion matrix of the BFO-ShuffleNet model for the four brain MRI classes.

Figure 8 illustrates the overall four-class confusion matrix of the BFO-ShuffleNet model. The model correctly classified 990 of the 1000 MRI images, giving an overall accuracy of 99.00%. Most samples were concentrated along the main diagonal, indicating that only a few images were assigned to the wrong class.

**Figure 9:**
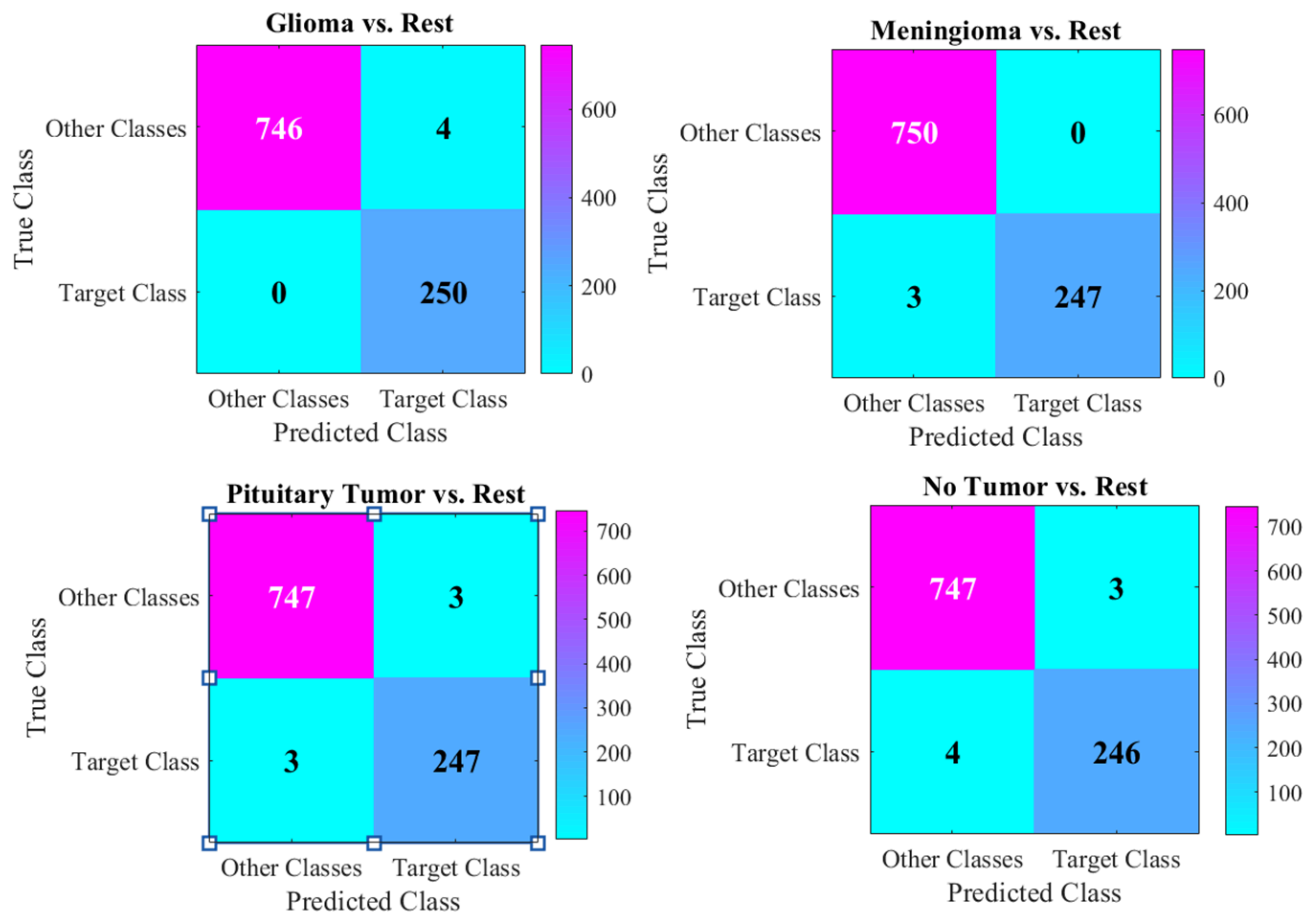
Class-wise one-versus-rest confusion matrices for the BFO-ShuffleNet model.

Figure 9 shows the one-versus-rest confusion matrices for the four diagnostic classes. The confusion matrices were generated from the out-of-fold predictions of one representative repetition of the repeated stratified five-fold cross-validation.

Glioma showed 100.00% sensitivity and 99.47% specificity. Meningioma achieved 98.80% sensitivity and 100.00% specificity. Pituitary tumor and the no-tumor class reached sensitivities of 98.80% and 98.40%, respectively, while both classes achieved 99.60% specificity.

Table 5 summarizes the class-wise classification performance of the proposed BFO–ShuffleNet model. The table reports TP, TN, FP, FN, sensitivity, specificity, precision, F1-score, and AUC for each diagnostic class.

Sensitivity ranged from 98.40% to 100.00%, while specificity ranged from 99.47% to 100.00%. Glioma achieved 100.00% sensitivity, and no false-positive predictions were observed for meningioma. High precision, F1-score, and AUC values were obtained for all four classes.

**Figure 10:**
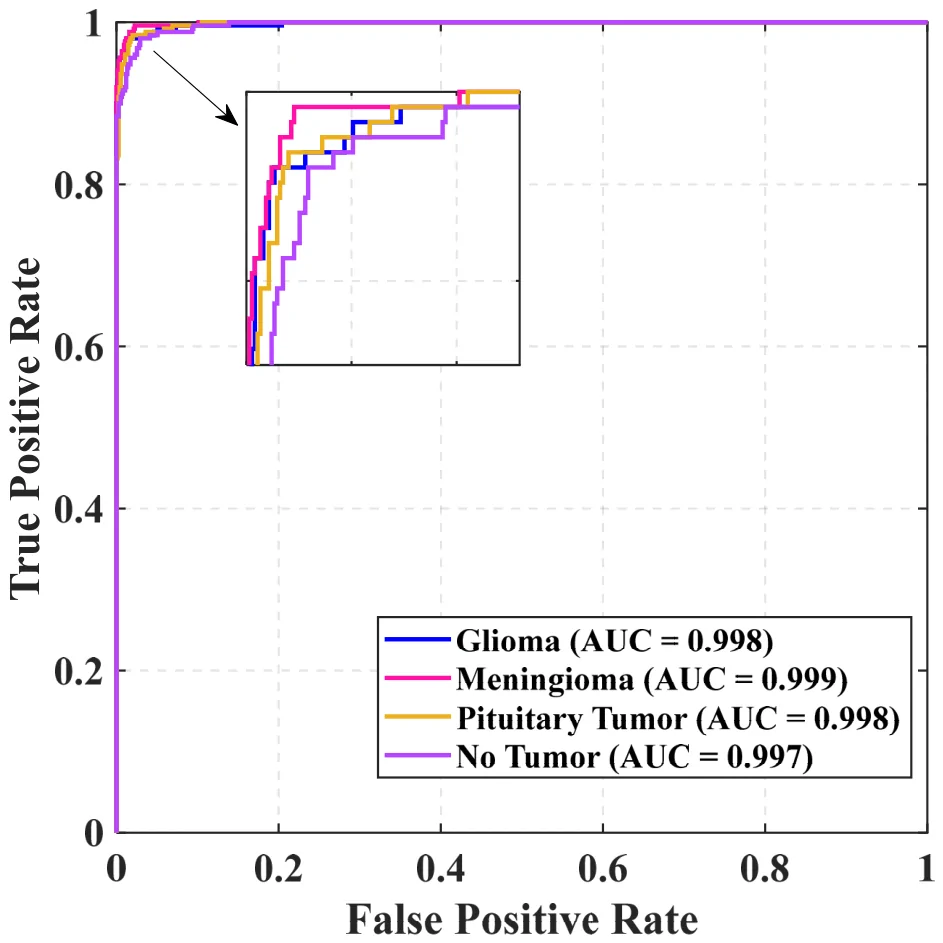
One-versus-rest ROC curves of the proposed BFO–ShuffleNet model for the four brain MRI classes.

Figure 10 presents the one-versus-rest ROC curves for the BFO-ShuffleNet model. Each ROC curve was obtained by treating one class as the positive class and the remaining three classes as the negative class. All four curves are concentrated near the upper-left region of the ROC space, showing good separation between the target class and the other classes. The AUC values are 0.998 for glioma, 0.999 for meningioma, 0.998 for pituitary tumor, and 0.997 for the no-tumor class.

The proposed model was evaluated on the public Kaggle Brain MRI Dataset using stratified five-fold cross-validation. The training and validation subsets were separated in each fold to prevent information leakage. However, no independent external dataset was used in this study. Therefore, the reported results represent internal validation on the selected dataset. External validation on an independent MRI dataset is required to assess the generalizability of the proposed model.

## 5. Conclusions

This research uses pretrained CNN features, BFO, and a multiclass SVM for brain MRI classification. BFO improved the classification results for VGG16, VGG19, InceptionV3, and ResNet50, with the highest scores obtained from the VGG16-BFO combination. The BFO-ShuffleNet model was also evaluated against PSO-Xception, WOA-MobileNet, GA-Inception, and GWO-EfficientNet under identical experimental conditions. It achieved an accuracy of 99.00 ± 0.12%, an F1-score of 98.99 ± 0.14%, and an AUC of 99.21 ± 0.13% while selecting 452 ± 19 features. Statistical tests showed significant differences between BFO–ShuffleNet and the other methods. These findings apply only to the MRI dataset and experimental setup used in this study. Additional evaluation on independent external datasets is required before the method can be assessed for routine clinical use. Future work will validate the proposed framework on independent multi-center MRI datasets using patient-level data separation and cross-dataset testing. Explainable AI methods will also be investigated to clarify the image regions that influence the classification decision.

## Figures and Tables

**Table 1:** Experimental protocol.

Parameter	Value
Dataset	Public Kaggle Brain MRI Dataset
Number of categories	4
Classes	Glioma, meningioma, pituitary tumor, no tumor
Input image size	224 × 224
Image normalization	Pixel/255
Label encoding	One-hot
CNN backbones used in BFO ablation study	VGG16, VGG19, InceptionV3, and ResNet50
CNN backbones used in final CNN–optimizer comparison	Xception, MobileNet, Inception, EfficientNet, and ShuffleNet
Feature extraction	Deep features extracted from pretrained CNN models
Feature selection	Bitterling Fish Optimization (BFO)
Validation strategy	Stratified 5-fold cross-validation
Training ratio	80%
Validation ratio	20%

**Table 2:** Implementation parameters for BFO and other optimization algorithms.

Parameter	BFO	PSO	GA	GWO	WOA
Population size	30	30	30	30	30
Maximum iterations	100	100	100	100	100
Number of independent runs	30	30	30	30	30
Search space	Binary	Binary	Binary	Binary	Binary
Feature representation	Binary solution vector	Binary solution vector	Binary solution vector	Binary solution vector	Binary solution vector
Initialization	Uniform random	Uniform random	Uniform random	Uniform random	Uniform random
Random seed	42	42	42	42	42

**Table 3:** Performance comparison of classifiers using BFO-selected VGG16 features.

Classification Method	Sensitivity (%)	Specificity (%)	Precision (%)	Accuracy (%)	Process Time (Sec.)
ANN	97.2	97.9	97.9	97.55	0.03
SVM	97.33	98.37	98.38	97.84	0.01
Decision Tree	96.28	98.23	98.23	97.25	0.01
AdaBoost	98.13	97.11	97.17	97.63	0.03
Random Forest	96.77	97.58	97.59	97.17	0.03
Ensemble	96.74	97.9	97.92	97.31	0.04
BFO-ShuffleNet	97.81	96.55	96.47	97.17	0.01

**Table 4:** Statistical comparison of the evaluated CNN–optimizer models.

CNN–Optimizer Model	Accuracy (%)	95% CI	F1-Score (%)	AUC (%)	Selected Features	Time (s)	Holm-Adjusted *p*-Value vs. BFO
PSO-Xception	97.22 ± 0.36	97.12–97.32	97.39 ± 0.32	96.80 ± 0.32	684 ± 33	85.7 ± 4.2	<0.001
WOA-MobileNet	98.24 ± 0.23	98.18–98.30	98.56 ± 0.23	97.67 ± 0.23	572 ± 22	79.4 ± 3.8	<0.001
GA-Inception	98.58 ± 0.24	98.51–98.65	98.72 ± 0.24	98.76 ± 0.24	525 ± 24	91.9 ± 5.5	0.002
GWO-EfficientNet	98.89 ± 0.18	98.84–98.94	98.70 ± 0.18	99.08 ± 0.16	499 ± 22	75.8 ± 3.4	0.018
BFO-ShuffleNet	99.00 ± 0.12	98.97–99.03	98.99 ± 0.14	99.21 ± 0.13	452 ± 19	69.9 ± 2.9	—

**Table 5:** Class-wise classification performance of the BFO-ShuffleNet model.

Class	TP	TN	FP	FN	Sensitivity (%)	Specificity (%)	Precision (%)	F1-Score (%)	AUC
Glioma	250	746	4	0	100	99.47	98.43	99.21	0.998
Meningioma	247	750	0	3	98.8	100	100	99.4	0.999
Pituitary Tumor	247	747	3	3	98.8	99.6	98.8	98.8	0.998
No Tumor	246	747	3	4	98.4	99.6	98.8	98.6	0.997

## Data Availability

The original contributions presented in this study are included in the article. Further inquiries can be directed to the corresponding author.
